# CHALLENGING AGEISM IN PSYCHOLOGY EDUCATION: THE ROLE OF GEROPSYCHOLOGY COURSES AMONG PSYCHOLOGY STUDENTS

**DOI:** 10.1093/geroni/igad104.2696

**Published:** 2023-12-21

**Authors:** Verena Kleissner, Tim Kuball, Georg Jahn

**Affiliations:** Chemnitz University of Technology, Chemnitz, Sachsen, Germany; Chemnitz University of Technology, Chemnitz, Sachsen, Germany; Chemnitz University of Technology, Chemnitz, Sachsen, Germany

## Abstract

Ageism includes negative attitudes, stereotyping, and discriminatory behavior toward older adults and is associated with negative consequences for health and well-being of the growing older population. In addition, ageism affects structural health care including psychological treatment of older individuals. Since educational interventions have been shown to be effective in reducing ageist prejudice and improving knowledge about older people, courses in gerontology taught as part of the psychology curriculum might help future psychologists to be better equipped to challenge ageism and to provide more effective and compassionate care to older adults. In the present study, we compared psychology students (n = 50) that chose courses in applied geropsychology with students of other specialization of choice (n = 26) on multiple scales including affective (attitudes), cognitive (stereotypes) and behavioral (discrimination) dimensions. Questionnaires were completed at the beginning and the end of one semester to conduct a within pre- and post-treatment assessment and analysis with a comparison group. Results showed that geropsychology students improved in self-reported knowledge about aging and older adults (d = 0.71). However, other tests did not show a statistically reliable improvement on the three assessed ageism dimensions within one semester. Findings implicate that gained knowledge about aging does not necessarily lower prejudice. In our discussion we highlight possible interventions targeting psychology students and reflect on methods to assess attitudes, stereotypes, and behavioral aspects of ageism in participants with high education level.

